# Induced pluripotent stem cell-derived smooth muscle cells to study cardiovascular calcification

**DOI:** 10.3389/fcvm.2022.925777

**Published:** 2022-07-22

**Authors:** Samantha K. Atkins, Abhijeet R. Sonawane, Romi Brouwhuis, Johana Barrientos, Anna Ha, Maximillian Rogers, Takeshi Tanaka, Takehito Okui, Shiori Kuraoka, Sasha A. Singh, Masanori Aikawa, Elena Aikawa

**Affiliations:** ^1^Center for Interdisciplinary Cardiovascular Sciences, Division of Cardiovascular Medicine, Brigham and Women’s Hospital, Harvard Medical School, Boston, MA, United States; ^2^Center for Excellence in Vascular Biology, Department of Medicine, Brigham and Women’s Hospital, Harvard Medical School, Boston, MA, United States

**Keywords:** calcification, cardiovascular diseases, induced pluripotent stem cells, smooth muscle cells (SMCs), proteomics and bioinformatics, stem cell differentiation and reprogramming

## Abstract

Cardiovascular calcification is the lead predictor of cardiovascular events and the top cause of morbidity and mortality worldwide. To date, only invasive surgical options are available to treat cardiovascular calcification despite the growing understanding of underlying pathological mechanisms. Key players in vascular calcification are vascular smooth muscle cells (SMCs), which transform into calcifying SMCs and secrete mineralizing extracellular vesicles that form microcalcifications, subsequently increasing plaque instability and consequential plaque rupture. There is an increasing, practical need for a large scale and inexhaustible source of functional SMCs. Here we describe an induced pluripotent stem cell (iPSC)-derived model of SMCs by differentiating iPSCs toward SMCs to study the pathogenesis of vascular calcification. Specifically, we characterize the proteome during iPSC differentiation to better understand the cellular dynamics during this process. First, we differentiated human iPSCs toward an induced-SMC (iSMC) phenotype in a 10-day protocol. The success of iSMC differentiation was demonstrated through morphological analysis, immunofluorescent staining, flow cytometry, and proteomics characterization. Proteomics was performed throughout the entire differentiation time course to provide a robust, well-defined starting and ending cell population. Proteomics data verified iPSC differentiation to iSMCs, and functional enrichment of proteins on different days showed the key pathways changing during iSMC development. Proteomics comparison with primary human SMCs showed a high correlation with iSMCs. After iSMC differentiation, we initiated calcification in the iSMCs by culturing the cells in osteogenic media for 17 days. Calcification was verified using Alizarin Red S staining and proteomics data analysis. This study presents an inexhaustible source of functional vascular SMCs and calcifying vascular SMCs to create an *in vitro* model of vascular calcification in osteogenic conditions, with high potential for future applications in cardiovascular calcification research.

## Introduction

Cardiovascular calcification is the lead predictor of adverse cardiovascular events, which has become the top cause of morbidity and mortality worldwide (1–3). Despite the increasing understanding of the underlying pathological mechanisms, to date there are no available pharmacological treatments to slow or stop the calcification process (4). Key players in vascular calcification are vascular smooth muscle cells (SMCs). In addition, immune cells, including macrophages, have been recently described to play a role (5–7). SMCs transform into calcifying SMCs and secrete mineralizing extracellular vesicles that form microcalcifications, subsequently increasing plaque instability and consequential plaque rupture (8, 9). Vascular disease susceptibility is hypothesized to be dependent on SMC germ layer origin, with vascular SMCs originating from all three germ layers. SMCs that contribute to calcification are thought to originate from the lateral plate mesoderm germ layer (10). On top of heterogeneity in origin, vascular SMCs also show heterogeneity in phenotype, and show phenotype plasticity as a response to environmental cues (11).

Experimental models for unraveling calcification mechanisms include human tissue samples, cell culture and animal models. The large biological differences between species, together with the limited proliferative capacity and donor to donor variability of primary SMCs in culture, make data analysis and statistical comparisons incredibly challenging (12). Primary human SMCs, like many other primary cell types, are limited by their capacity to withstand multiple passages *in vitro* and display passage-dependent expression of SMC marker proteins (13, 14). There is a significant need for large-scale, reproducible lines of SMCs to study disease mechanisms and offer potential non-invasive, therapeutic solutions. This unmet research need necessitates the development of a standardized source of human SMCs for two important reasons: (1) to create a human tissue model for studying the pathological mechanisms underlying vascular calcification; and (2) to subsequently discover novel therapeutic targets for treatment of vascular calcification. One approach to resolve the issues with passage-dependency and limited proliferative capacity in primary human cell lines is to generate such a disease model is by using human iPSCs for *in vitro* studies and screening. iPSCs can be used to create an alternative source of cell lines for disease modeling, tissue engineering, and drug screening. In this paper, we present a method to create iPSC-derived induced SMCs (iSMCs) that can calcify under osteogenic conditions to represent the disease model of atherosclerosis. In addition, we carefully defined the proteome at every stage throughout the differentiation timeline. Most previous studies of iPSC-derived SMCs have poorly defined starting cell populations with a range of phenotypically diverse iSMC end products (15). In this model, we have adapted the approach by Patsch et al., to create an inexhaustible line of iSMCs (16). We have characterized the population throughout the differentiation timeline using systems biology approaches, and we have gone a step further to recapitulate SMC calcification *in vitro*.

## Materials and methods

### Cell culture

Human induced pluripotent stem cells (iPSCs) derived from foreskin fibroblasts of one single donor (BJiPSCs, ATCC) were used in this study. iPSCs were cultured in mTeSR™ Plus medium (StemCell Technologies), enriched with 1% antibiotic-antimycotic (Anti-Anti, Gibco), on plates coated with Matrigel matrix (50–100 μg/ml) (Fisher Scientific). iPSCs were passaged at 70–80% confluency using ReLeSR™ (StemCell Technologies) and replated as aggregates at a dilution of 1:20. At the initial thaw, before culturing in mTeSR™ Plus medium, cells were cultured on Matrigel-coated plates in mTeSR™ Plus medium enriched with 10 μM ROCK inhibitor Y-27632 (StemCell Technologies) for 24 h. iPSCs were stored at −180°C as aggregates using mFreSR™ (StemCell Technologies), or as single cells using FreSR-S (StemCell Technologies). Each experiment was carried out in triplicate.

For flow cytometry and proteomic comparison of our iSMCs to primary SMCs, human coronary artery smooth muscle cells (hCASMCs, Promocell, three donors) were cultured in SMC Growth Medium 2 (Promocell) supplemented with epidermal growth factor (0.5 ng/ml), insulin (5 μg/ml), basic fibroblast growth factor-B (2 ng/ml), 10% fetal bovine serum and 1% penicillin-streptomycin (P/S). For flow cytometry analysis, human umbilical vein cells (HUVECS, Lonza, one donor) were used as a negative control. HUVECs were cultured in EBM-2 Basal medium, enriched with EGM-2 supplements (Lonza) and 1% P/S. All primary cells were passaged using 0.25% Trypsin-EDTA (ATCC) and counted using Countess II (Life Technologies).

### Study design and sample replicates

Induced pluripotent stem cell differentiation was carried out in three independent experiments (*n* = 3) using one single donor (BJiPSCs, ATCC) due to limited availability of commercially available donors from ATCC. For comparison to primary hCASMCs under normal and osteogenic conditions, three separate donors (*N* = 3) obtained from Promocell were used.

### Induced pluripotent stem cell-induced-smooth muscle cell differentiation

For differentiation, a protocol was set up based on a previous study that reported successful differentiation of iPSCs to iSMCs (16). Three differentiations were performed on iPSCs between passages 6 and 9 from one single donor. On day 0 iPSCs were cultured as described above (see section “Cell Culture”) in mTeSR™ Plus, on Matrigel-coated plates. Upon passaging, iPSCs were plated on Matrigel coated plates at a density of 40,000 cells/cm^2^ in mTeSR™ Plus, enriched with 10 μM ROCK inhibitor Y27632. From here on N2B27 medium was used for culturing the cells. N2B27 consists of DMEM/F12 with 15 mM Hepes (StemCell Technologies) and Neurobasal medium (Life Technologies) at a ratio of 1:1, supplemented with 2% B27 minus vitamin A (Life Technologies), 1% N2 supplement (Life Technologies), 1% antibiotic-antimycotic solution and 0.1% β-mercaptoethanol (Life Technologies). Twenty-four hours after the iPSCs were plated, the cells were cultured in mesoderm induction media, consisting of N2B27, enriched with 8 μM CHIR99021 (StemCell Technologies) and 2.5 ng/ml BMP4 (Peprotech). The lower amount of BMP4 was the only modification to the original protocol by Patsch et al. Mesoderm induction was carried out for 3 days with no media change. At day 3 the media was replaced with SMC inducing media, containing N2B27 supplemented with 10 ng/ml PDGF-BB (Peprotech) and 2 ng/ml ActivinA (Peprotech). On day 5, the cells were removed with trypsin-EDTA (0.25%, ATCC), washed in N2B27 media and pelleted by centrifugation (150 × gravity, 5 min). To induce SMC maturation, the cells were resuspended in N2B27 media enriched with 2 μg/ml heparin (StemCell Technologies) and 2 ng/ml ActivinA (Peprotech) and plated on collagen coated wells (Corning Biocoat) at a density of 30,000 cells/cm^2^. The cells were cultured with regular media changes until day 10, at which the differentiation media was replaced with SMC Growth Media 2 (SMGM2) (Promocell). Further expansion and culturing of the cells was done in SMGM2. An in-depth protocol can be found in Supplementary Material 1.

### Osteogenic differentiation of induced-smooth muscle cells into calcifying induced-smooth muscle cells

Osteogenic differentiation of iSMCs was carried out three times (*n* = 3, 1 donor). For the osteogenic differentiation of iSMCs into calcifying (c)-iSMCs, the iSMCs were plated on 24-well plates at a density of 100,000 cells/well in SMGM2. After 24 h, calcification was induced by culturing the cells in osteogenic medium (OM), containing GlutaMAX™ DMEM, 10% FBS and 1% penicillin-streptomycin (termed normal media, NM), with addition of osteogenic factors; 10 mM dexamethasone (Fisher Scientific), 10 mM β-glycerol phosphate (Millipore Sigma) and 100 μM L-ascorbic acid 2-phosphate (Millipore Sigma). Media was refreshed every 3 days.

### Immunocytochemistry

Cells were fixed in 4% paraformaldehyde (Sigma-Aldrich) for 10 min, followed by a 5-min permeabilization step with 100% methanol, washed twice with PBS for 5 min per wash. Next, the cells were blocked with 0.1% Tween (Sigma-Aldrich) and 10% normal donkey serum blocking solution (Sigma-Aldrich) in PBS at room temperature. Cells were incubated overnight at 4°C with primary antibodies; dilution of 1:100 rabbit anti-αSMA (Abcam #ab5694), 1:100 rabbit anti-Nanog (Abcam #ab21624), and 1:250 mouse anti-smMHC11 (Abcam #ab683). The cells were then incubated for 1 h at room temperature with secondary antibodies donkey anti-mouse 488 (Thermo Fisher Scientific #a21202) and donkey anti-rabbit 594 (Thermo Fisher Scientific #A21207) at 1:200 dilution. After rinsing, cells were incubated with a drop of NucBlue Fixed Cell Ready Probes Reagent (Thermo Fisher Scientific #R37606) in PBS for 5 min. Cells were stored at 4°C in the dark. Examination was done using the confocal microscope A1 (Nikon Instruments Inc), and all images were processed with Elements 3.20 software (Nikon Instruments Inc).

### Flow cytometry

For flow cytometry sample preparation, the cell samples were harvested and collected in EasySep buffer (StemCell Technologies) at a concentration of 10 × 10^6^ cells/ml. The samples were incubated for 20 min in the dark on ice with antibodies Alexa fluor 647 mouse anti-human TRA-1-60 (BD Biosciences), FITC mouse anti-human TRA-1-81 (BD Biosciences), PE mouse anti-human CD140b (BD Biosciences), and APC-Cy7 mouse anti-human CD31 (BD Biosciences). Next, the cell samples were diluted ten times in EasySep buffer, and centrifuged [350 × *g* × 5 min]. Supernatant was removed and the dilution step was repeated. After supernatant was removed, the cell samples were resuspended in EasySep buffer at a concentration of 3.33 × 10^6^ cells/ml and filtered through a round-bottom tube with cell strainer (Falcon). The stained cell samples were analyzed using the BD FACSAria™IIu machine and the accompanied BD FACSDiva software. Acquisition of 10,000 events was made, and data analysis was performed after gating for single cell population minus cell debris.

### Proteomics sample preparation

Approximately 1 × 10^6^ cells from a 6-well plate were collected in 400 μl of RIPA lysis buffer with protease inhibitor cocktail (Thermo Fisher Scientific) according to the manufacturer’s protocol. There are two datasets. Dataset 1 consists of the iPSCs differentiated into iSMCs (*n* = 3, 1 donor, time points: day 0, day 3, day 5, day 7, day 10) and a Matrigel negative control “Mgel”; Supplementary Material 2 (Table 1). Dataset 2 consists of the iSMCs cultured in NM and OM collected at days 10 and 17 (*n* = 3, 1 donor, time points: day 10 and day 17) as well as SMCs in NM and OM from day 14 in culture (3 donors, time point: day 14); Supplementary Material 3 (Table 2). The sampling time points for dataset 1 during iPSC to iSMC differentiation correspond with the specific induction protocols. Day 0 represents naïve iPSCs; day 3 represents lateral mesoderm; day 5 represents the start of SMC induction; day 7 represents a midpoint for iSMC purification; day 10 represents the endpoint for iSMC differentiation. Dataset 2 timespoints are consistent with when calcification is present as indicated by Alizarin Red staining; days 10 and 17 for iSMCs and day 14 for primary human coronary artery SMCs.

The protein content was determined by the Pierce BCA assay (Thermo Fisher Scientific). A volume equivalent to 20 μg of protein was used for further processing. Next, the samples were prepared using iST Sample Preparation Kit (PreOmics, Germany) according to the protocol provided by manufacturer, without sonication and with a 90 min incubation at 37°C. The samples processed by the iST kit were resuspended in 40 μl of LC-LOAD (PreOmics).

### Mass spectrometry

Data-dependent acquisitions (DDAs, unbiased peptide sampling)–the peptides were and analyzed using the Orbitrap Fusion Lumos Tribrid mass spectrometer (Thermo Fisher Scientific) fronted with an EasySpray ion source, and coupled to an Easy-nLC1000 HPLC pump (Thermo Fisher Scientific). The peptides were separated using a dual column set-up: an Acclaim PepMap 100 C18 trap column, 75 μm × 20 mm; and a PepMap RSLC C18 EASY-Spray LC heated (45°C) column, 75 μm × 250 mm (Thermo Fisher Scientific). The gradient flow rate was 300 nl/min from 5 to 21% solvent B (acetonitrile/0.1% formic acid) for 75 min, 21 to 30 % Solvent B for 15 min, followed by 10 min of a “jigsaw wash,” alternating between 5 and 95 % Solvent B. Solvent A was 0.1% formic acid. The instrument was set to 120 K resolution, and the top N precursor ions in a 3 s cycle time (within a scan range of 375–1,500 m/z; isolation window, 1.6 m/z; ion trap scan rate, normal) were subjected to collision induced dissociation (collision energy 30%) for peptide sequencing (or MS/MS). Dynamic exclusion was enabled (60 s).

### Mass spectrometry/mass spectrometry data analysis

The MS/MS spectra were queried against the human (downloaded on November 21, 2018; 155,133 entries) using the HT-SEQUEST search algorithm, *via* the Proteome Discoverer (PD) Package (version 2.2, Thermo Fisher Scientific). Methionine oxidation and n-terminal acetylation were set as a variable modifications, and carbamidomethylation of cysteine was set as a fixed modification. Peptides were filtered based on a 1% false discovery rate (FDR) (17) based on the reverse database (decoy) results (18, 19). In order to quantify peptide precursors detected in the MS1 but not sequenced from sample to sample (across mass spectrometric samples in Supplementary Material 2 (Table 1)), we enabled the “Feature Mapper” node. Chromatographic alignment was done with a maximum retention time (RT) shift of 10 min and a mass tolerance of 10 ppm. Feature linking and mapping settings were, RT tolerance minimum of 0 min, mass tolerance of 10 ppm and signal-to-noise minimum of five. Precursor peptide abundances were based on their chromatographic intensities and total peptide amount was used for normalization. Peptides assigned to a given protein group, and not present in any other protein group, were considered as unique. Consequently, each protein group is represented by a single master protein (PD Grouping feature). We used unique and razor peptides per protein for quantification.

### Proteomics dataset processing

In house scripts written in Python v3.4 (20) were used to replace missing values with zero values and to perform per-sample median normalization on master protein (more than 2 unique peptides) intensities as quantified by PD. Dataset 1 (Supplementary Material 2 (Table 1)) was used to analyze the differentiation time course. Dataset 2 (Supplementary Material 2 (Table 2)) compares the protein expression from iSMCs in NM and OM at two time points (day 10 and day 17; 1 donor, *n* = 3) to primary human coronary artery SMCs in NM and OM (day 14; 3 donors).

### High dimensional clustering of protein abundances

Analysis of proteome abundance patterns over the 10 day differentiation time course was performed using a high-dimensional clustering software, XINA^1^ (21). The median-normalized abundance of proteins for three independent differentiation experiments (*n* = 3, 1 donor) were combined into a single dataset for subsequent clustering analysis by time point. Clusters differentiating peak protein abundance per time point (days 0, 3, 5, 7, and 10) were prioritized. The top ten most abundant shared proteins per time point were normalized by the daily expression and plotted with labels indicating the most enriched gene ontological terms.

### Day-specific proteomics analysis

The proteomics data were used to obtain the mean abundance values for each protein for days 0, 3, 5, 7 and 10 separately (Supplementary Figure 1). To obtain the day-specific proteins, we compared the mean abundance level ${\left\langle p\right\rangle }_{j}^{d}$ of a protein j and at day d to the mean ⟨*p*⟩^*all*^ and standard deviation σ of all the *p^all^* on all days:


$$Z\,s\,c\,o\,r\,e\,A\,b\,u\,n\,d\,a\,n\,c\,e\,p_{j}^{d}=\frac{\left({\left\langle p\right\rangle }_{j}^{d}-{\left\langle p\right\rangle }^{a\,l\,l}\right)}{\sigma }.$$

We define a protein as specific to day d if the z-score abundance is $p_{j}^{d}>1.5$. The threshold 1.5 was chosen such that there is a balance between number of proteins specific to at least one day (Supplementary Figures 1B,C). This definition allowed us to utilize the full distribution of protein abundance values. The list of proteins specific to each day is given in Supplementary Material 2 (Table 3).

To investigate the functional enrichment of day-specific proteins, we acquired Gene Ontology (GO) terms for each group of proteins using R (v 4.0.3) package clusterProfiler (v 3.18) (22) with adjusted *p*-value < 0.05 (Benjamini-Hochberg) and used AnnotationDbi R package “org.Hs.eg.db” (v 3.12) to map gene identifiers.

To perform differential abundance analysis on the proteomics data between adjacent days, we used limma (3.46) package in R (v 4.0.3) with voom transformation to remove mean variance dependence and Benjamini-Hochberg for adjusted *p*-value. The volcano plots were plotted using R package EnhancedVolcano (v 1.8) with adjusted *p*-value < 10e-5 and abs(logFC) > 1 shown in color.

### Alizarin red staining for smooth muscle cell calcification

Cells were stained with Alizarin Red S (Sigma Aldrich) to analyze calcium deposition. Cells were washed with PBS before being fixed with 10% formalin solution for 10 min. Afterward the cells were washed three times with distilled water. Pictures were taken using a scanner (Ricoh Aficio MP 3500) at 300 dpi.

### Statistical analyses

Data was statistically analyzed using *t*-test or ANOVA with *post-hoc* tests using GraphPad PRISM or Qlucore Omics Explorer. ImageJ, Adobe Photoshop and Adobe Illustrator were used to process and present the images and graphs, and to create the figures. Pearson correlation was computed using cor() function in R.

## Results

### Induced pluripotent stem cells undergo dynamic morphological changes while transitioning to induced-smooth muscle cells

**FIGURE 1:**
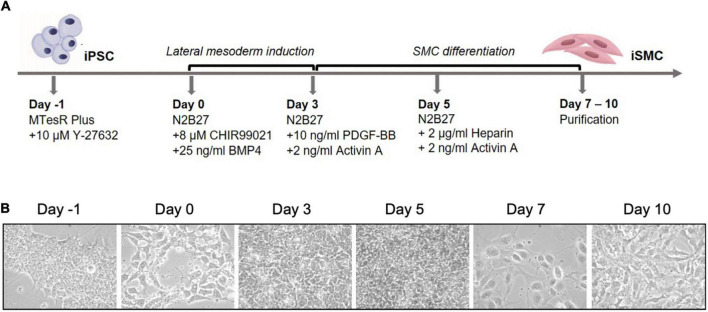
Differentiation timeline of iPSCs into iSMCs. **(A)** Schematic of the differentiation timeline and protocol. **(B)** Brightfield microscopy images of the differentiation time course from iPSCs to mesoderm and then to iSMCs (magnification = 20×). Representative images of *n* = 3 differentiation replicates.

Induced pluripotent stem cells were induced into mesoderm and then SMCs. Arteries arise from the lateral plate mesoderm germ layer, and this germ layer is hypothesized to be the origin of SMCs near areas of calcification (10). To achieve a population of iSMCs capable of recapitulating the calcification process, the iPSCs were transitioned into mesoderm from day 0 to day 3, before beginning SMC differentiation. The differentiation timeline (Figure 1A) to induce the iPSCs into a mesoderm germ layer, and then conditioned the cells with combinations of PDGF-BB, activin A, and heparin to induce the iSMC phenotype (16). Brightfield microscopy at 20 × resolution on days: −1, 0, 3, 5, 7, and 10 demonstrate the morphological changes that take place over the differentiation time course (Figure 1B). The cells begin as iPSC colonies with defined edges (day −1) that were replated as single cells with ROCK inhibitor, and at day 0 showed the presence of small colonies with the absence of smooth and defined colony edges, typical of iPSCs treated with ROCK inhibitor (Y-27632). Over the 10-day differentiation time course, the cell morphology underwent dynamic changes that result in a stretched SMC phenotype by day 10 (Figure 1B).

### Differentiation protocol yields high efficiency of induced-smooth muscle cells by flow cytometry

**FIGURE 2:**
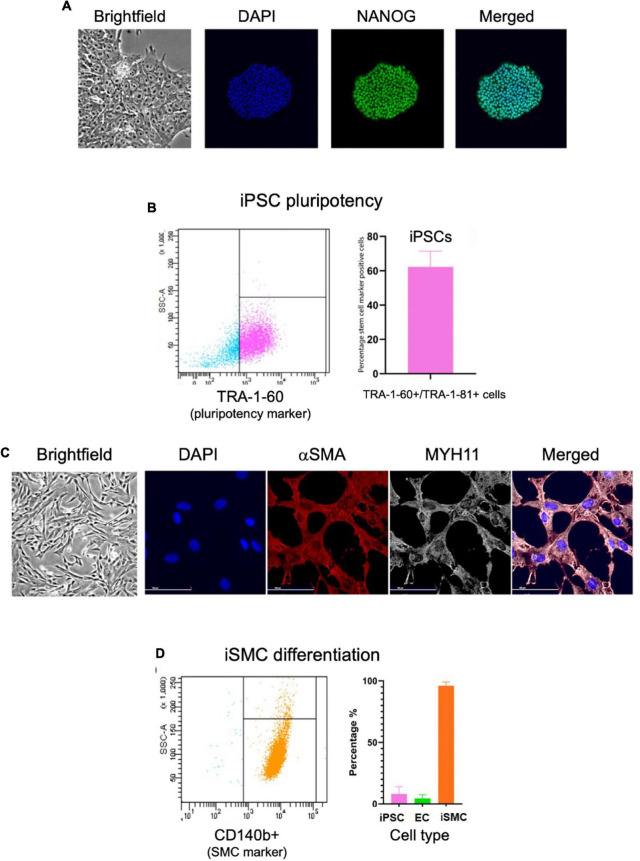
Characterization of iPSCs at day 0 and iSMCs at day 10. **(A)** Brightfield microscopy image (20× magnification) of iPSC colony morphology, and immunofluorescent staining (20× magnification) for DAPI (blue), pluripotency marker NANOG (green), and merged. **(B)** Flow cytometry analysis on iPSC pluripotency marker TRA-1-60; left side (blue dots) representative flow cytometry analysis, right side (pink dots) flow cytometry detected percentage of stem cell marker positive cells. **(C)** Brightfield microscopy of iSMCs at day 10. Immunofluorescent staining for DAPI (blue), αSMA (red), MYH11 (white) and merged. **(D)** Flow cytometry analysis on iPSC marker TRA-1-60, EC marker CD31, and iSMC marker CD140b; left side representative flow cytometry analysis, right side flow cytometry detected percentage of stem cell marker, EC marker, and SMC marker. *n* = 3 differentiation replicates; mean ± SEM; scale bars = 100 μm (20× magnification).

We then assessed iPSCs for the correct morphology and the expression of pluripotency markers using immunofluorescence and flow cytometry. The immunofluorescent analysis showed the iPSCs had compact colonies with well-defined edges and expressed the pluripotency marker NANOG (Figure 2A). Flow cytometry for pluripotency markers TRA-1-60 and TRA-1-81 indicated a 62% pluripotent cell population (Figure 2B). Immunofluorescent staining on day 10 for SMC markers alpha smooth muscle actin (αSMA) and myosin heavy chain 11 (MYH11) showed the clear presence of these markers (Figure 2C). Flow cytometry of the iSMCs at day 10 showed 96% (± 3.1%) positive for SMC membrane marker protein CD140b (PDGFRB) (Figure 2D). Endothelial cells (ECs) are a potential byproduct of this particular differentiation protocol (16), and the EC marker CD31 was used to verify the absence of these cells. As a negative control, flow cytometry analysis of iSMCs for EC marker CD31 and pluripotency markers TRA-1-60 plus TRA-1-81 showed low percentage (4.47 and 8% positive cells, respectively), indicative of a pure population of iSMCs (Figure 2D).

### Induced-smooth muscle cells immunofluorescence shows OCT4 expression throughout differentiation and appearance of smooth muscle cell markers at days 7 and 10

**FIGURE 3:**
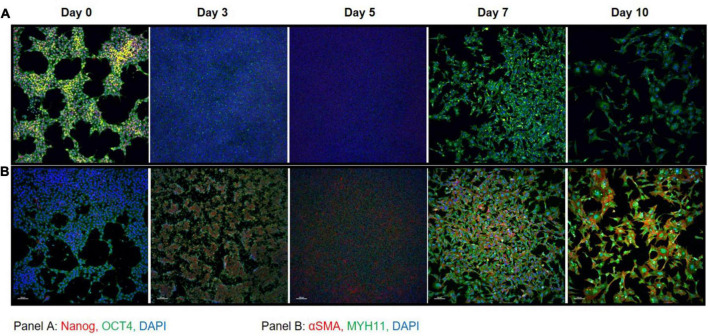
Immunocytochemistry on iPSC markers and SMC markers during differentiation. **(A)** iPSC markers NANOG (red) and OCT4 (green) with DAPI (blue) shows that OCT4 expression is maintained throughout the differentiation time course. **(B)** SMC markers αSMA (red) and MYH11 (green) with DAPI (blue) shows that by day 7 the cells express high levels of SMC marker proteins that persists through day 10. A total of 10× magnification, scale bars = 100 μm; representative images.

Immunofluorescent staining performed at days 0, 3, 5, 7, and 10 on iPSC markers (Figure 3A) and SMC markers (Figure 3B) during the differentiation time course demonstrated that cells lose NANOG expression while maintaining OCT4 in the perinuclear and cytosolic regions of the iSMCs. At days 7 and 10 these cells showed strong expression of SMC markers such as αSMA and MYH11 and displayed a spindle-shape morphology, characteristic of SMCs. This may explain why the flow cytometry analysis after differentiation showed a population of cells that are positive for both pluripotency marker (TRA-1-60+TRA-1-81) and SMC marker (CD140b), as some pluripotency expression remained at day 10.

### Induced pluripotent stem cell to induced-smooth muscle cell differentiation was confirmed by proteomics

**FIGURE 4:**
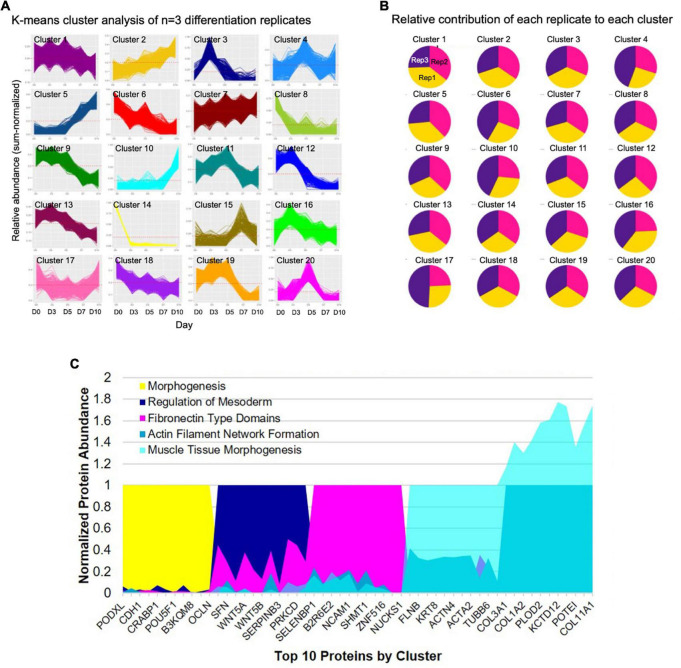
High-dimensional cluster analysis of the proteome describing the iPSC to iSMC transition. **(A)** K-means clustering analysis with 20 clusters describing the combined time-resolved protein abundances of *n* = 3 differentiation replicates. **(B)** Condition composition of the clusters shows equal distribution of each differentiation replicate in each cluster: yellow = replicate 1, pink = replicate 2, purple = replicate 3. **(C)** Expression of top 10 shared proteins between experimental replicates at each time point (day 0 = cluster 14; day 3 = cluster 3; day 5 = cluster 20; day 7 = cluster 5; day 10 = cluster 10) normalized by time point. String analysis on the shared proteins of each cluster was used to analyze GO terms.

A comprehensive characterization of the differentiation of iPSCs into iSMCs has not been done prior to this study. Previously, only prototypical markers of SMCs have been verified by Western blot at the end of the differentiation time course, but in this study, proteomics has allowed us to delve deeper into the molecular profiles throughout the differentiation process. To better understand the molecular changes along the differentiation trajectory, we collected proteomics data at five time points: Days 0, 3, 5, 7, and 10 (*n* = 3 differentiation replicates). First, we analyzed the data using a high-dimensional clustering tool XINA (21) to identify temporal patterns in protein expression/abundance using k-means clustering of the combined replicate data, resulting in 20 clusters (Figure 4A). Since this high-dimensional clustering method combined the three independent replicates, we could also confirm that each replicate exhibited similar time-resolved protein patterns since each cluster contains approximately same number of proteins from each replicate (Figure 4B).

A network of proteins co-expressed at the same time point is referred to as protein co-abundance and can be used to identify proteins that work together to perform a specific process or function (21). We identified clusters of co-abundant proteins at each stage of differentiation, namely, day 0 cluster number 14; day 3 cluster number 3; day 5 cluster number 20; day 7 cluster number 5, and day 10 cluster number 10 (Figure 4C). Day 0 notable proteins included POU5F, PODXL, and PROM1 and the top GO terms included tissue development and morphogenesis. Day 3 top GO terms indicated primitive streak formation, regulation of mesoderm development, and many other embryonic processes. Day 5 indicated fibronectin type domains. By day 7 GO terms indicated actin filament network formation, and day 10 showed collagen fibril organization, cell migration involved in sprouting angiogenesis, and muscle tissue morphogenesis, providing further evidence for successful differentiation into iSMCs.

### Induced pluripotent stem cell to smooth muscle cell differentiation is marked by a metabolic to a cell morphogenesis pathway transition

**FIGURE 5:**
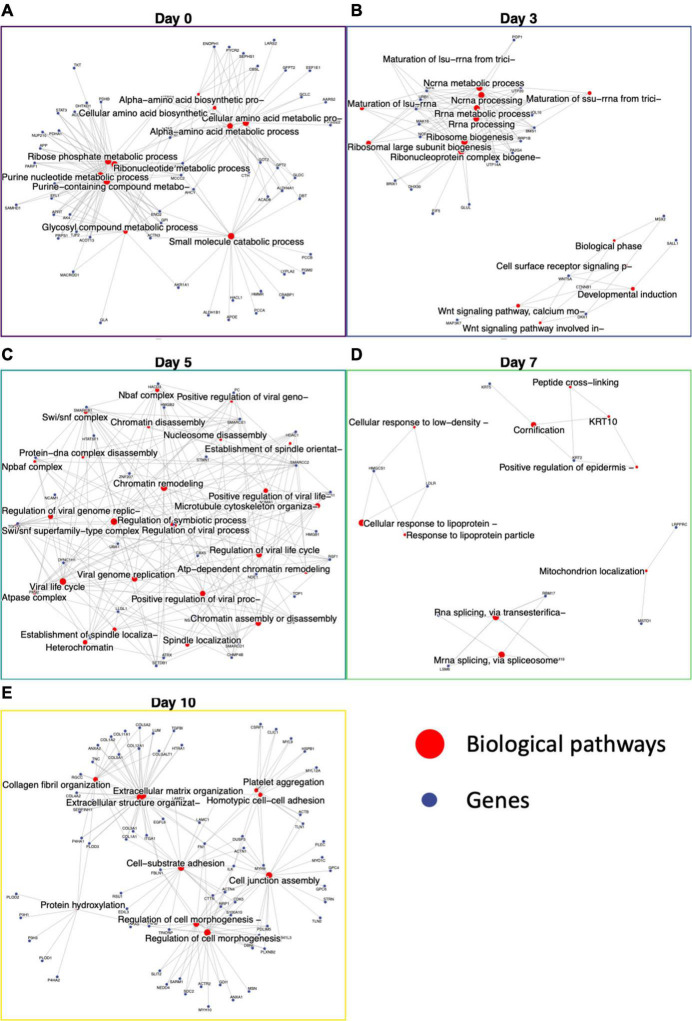
Functional enrichment of day-specific proteins accurately maps differentiation trajectory. Proteins were identified as day-specific with increase z-score abundance and function enrichment analysis was performed showing biological processes active on each day: Day 0, Day 3, Day 5, Day 7, and Day 10. **(A)** Day 0 major nodes include ribose phosphate and ribonucleotide metabolic processes. **(B)** Day 3 major nodes include RNA processing and Wnt signaling. **(C)** Day 5 nodes include chromatin assembly, disassembly, and remodeling. **(D)** Day 7 nodes include cornification and mitochondrion localization. **(E)** Day 10 nodes include extracellular matrix organization, regulation of cell morphogenesis, cell junction assembly, and cell-substrate adhesion.

Previous analysis using XINA showed general biological processes active at various days using selected protein clusters. To leverage the broad proteomics data and to look deeper into specific biological processes during the differentiation, we identified day-specific proteins using z-scored abundance (see section “Materials and Methods”). Using the differences in sample mean and global mean for all the proteins, we identified proteins with increased abundance on a particular day (Figure 5). For example, on day 0 we saw around 400 proteins with increased abundance such as PROM1, VSNL1, CDH1, SOX2, and POU5F1, involved in NADP metabolic and alpha-amino acid biosynthetic processes (Figure 5A). PROM1 modulates Rho/ROCK (23), CDH1 (E-cadherin) is required for iPSC pluripotency and self-renewal (24), and SOX2 and POU5F1 further confirmed pluripotency in our starting cell population (25). On Day 3, we found proteins MEST, VIM, and DDAH1, indicative of mesoderm formation (Figure 5B). MEST is known as the mesoderm-specific transcript, a negative regulator of adipogenesis (26). Vimentin upregulation is representative of mesoderm formation (27), and DDAH1 belongs to the mesoderm commitment pathway (28). The GO enrichment of 144 day 3 proteins included pathways involving RNA processing, developmental induction, ribosome biogenesis and primary lysosome.

After day 3, the cells were changed to SMC inducing media, and we saw proteins involved in chromatin remodeling, nucleosome assembly, nBAF and SWI/SNF complex (29) and establishment of spindle localization (Figure 5C). The first 5 days demonstrate the large switch in signaling that accompanies iPSC differentiation into iSMCs. On day 7, keratins 2 and 10 expression lead to functional enrichment of skin epidermis pathways, keratin filament pathway, mitochondrion localization, and intermediate filament cytoskeleton indicating structural formation (Figure 5D). Finally, proteins abundant at day 10 showed a robust SMC enrichment network, with pathways for actin filament organization, blood coagulation, cell morphogenesis, cell-substrate adhesion, and extracellular matrix organization that accompany successful iSMC differentiation (Figure 5E).

### Comparing proteomics from adjacent days to find activated and deactivated processes

**FIGURE 6:**
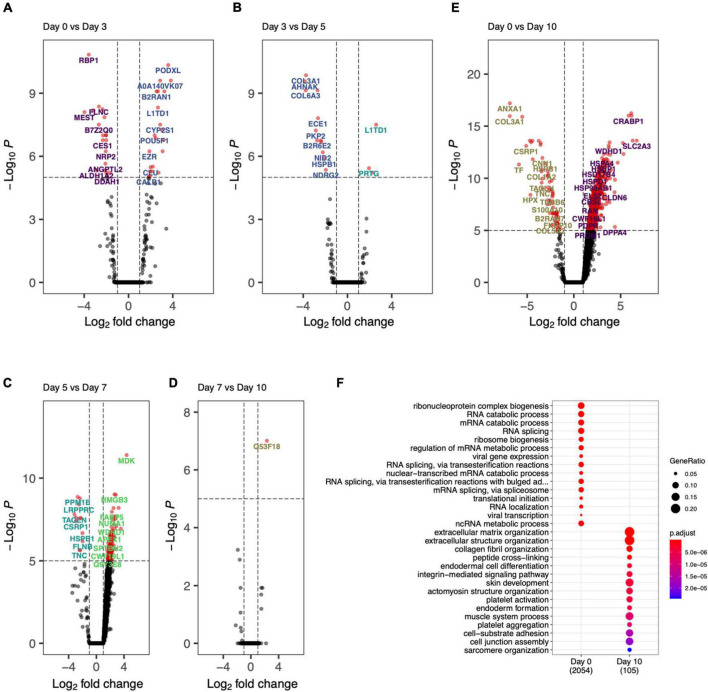
Day-to-day differential protein abundance analysis. Volcano plots of increased of previous day (shown on right with positive log fold change) vs. adjacent day (shown on left with -negative log fold change). **(A)** Day 0 vs. Day 3, **(B)** Day 3 vs. Day 5, **(C)** Day 5 vs. Day 7, **(D)** Day 7 vs. Day 10, **(E)** Day 0 vs. Day 10. **(F)** Bar chart showing the functional enriched processes on Day 0 and Day 10.

The day specific proteins showed protein abundances on particular days with respect to overall data. To probe the activation and deactivation of processes through-out the differentiation cycle, we performed differential abundance analysis between consecutive days. The volcano plots showed significantly enriched (adjusted *p*-value < 0.05 and abs(logFC) > 1) proteins between samples from adjacent days as well as end points (day 0 and day 10). Day 0 vs. day 3 (Figure 6A), we saw proteins PODXL, EZR, and DNMT3B indicative of the formation of apical actin-dependent microvilli and the establishment of DNA methylation patterns during development. Comparison of day 3 with day 0 and day 5 show significant enrichment in proteins VIM, MEST, COL6A1, L1TD1, and PRTG indicating processes like intermediate filament product, mesoderm-specific transcription, and fibronectin domains (Figure 6A). Protein enrichment at day 5, (Figure 6B) showed enhancement in collagens III and VI as well as MDK, SELENBP1, APEX1, ENPP1, and HEL-S-310. GO terms associated with these proteins include phosphodiesterase I activity and protein-containing complex binding. Interestingly, protein MDK is implicated in neointima formation after arterial injuries (30), and ENPP1 is required for SMC calcification (31). Differentially expressed proteins at day 7 (Figure 6C) included SMC marker proteins TAGLN and CNN1, as well as ANXA1, which is required for SMC calcification (7). The GO terms associated with these significantly enriched proteins include cellular response to vascular endothelial growth factor stimulus and positive regulation of blood vessel endothelial cell migration. There was one significantly enriched protein at day 7 as compared to day 10, which is unannotated protein Q53F18, a WH1 domain-containing family involved in actin polymerization^2^ (Figure 6D). This unannotated protein could represent a potential undiscovered SMC-specific protein required for differentiation.

Finally, by comparing day 0 to day 10 (Figures 6E,F) there was a clear decrease in stem cell marker proteins (DPPA4) and transcriptional regulation and elongation proteins (ENO2, DUT, PHF6, TCEA1). By looking at the proteins differentially abundant at day 10, SMC marker proteins COL1A1, ANXA1, CNN1, and ACTB implicated regulation and modulation of smooth muscle contraction. This analysis showed the cell differentiation protocol not only recapitulates robust iSMCs but also sheds light on trajectory of cellular evolution, which can potentially be used in modulating the protocol to evolve different SMC subtypes.

### Induced pluripotent stem cell-derived induced-smooth muscle cells show potential to calcify and have a high proteomic overlap with primary smooth muscle cells

**FIGURE 7:**
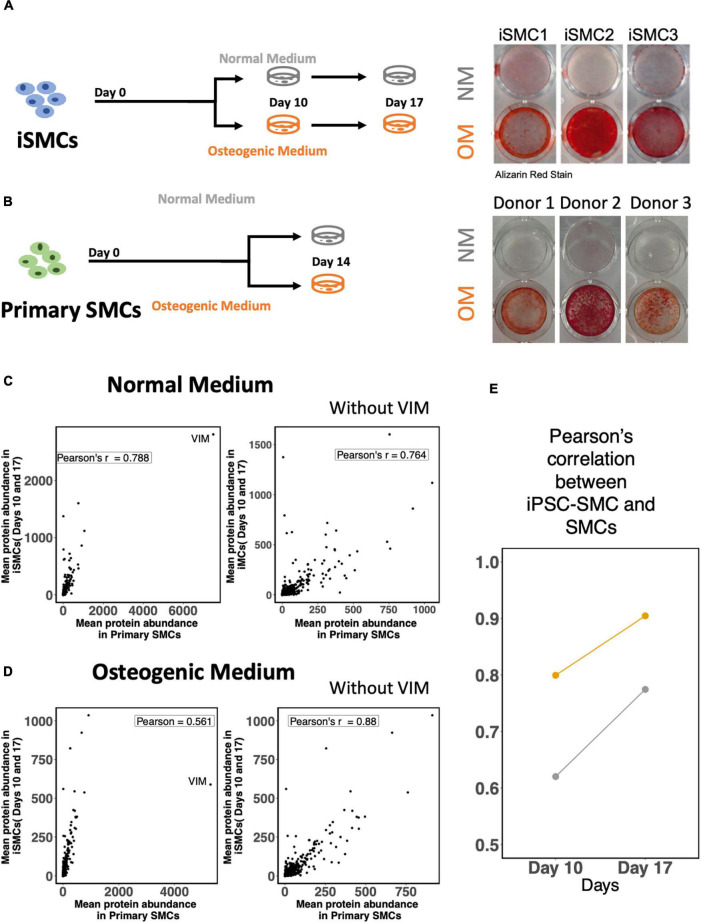
Induced-smooth muscle cells (iSMCs) calcify under osteogenic media (OM) conditions. **(A)** Study design and Alizarin Red S staining on iSMCs cultured in normal media (NM) and osteogenic media (OM) during three independent differentiations on Days 10 and 17. Bright red staining under OM conditions represents calcification in OM while no staining is observed in NM indicative of no calcification. **(B)** Study design and representative images of Alizarin Red S staining on primary SMCs cultured in normal media (NM) and osteogenic media (OM). **(C)** Pearson’s correlation between mean protein abundance between iSMCs and primary SMCs cultured with and without the inclusion of the protein vimentin in the analyses under NM. **(D)** Similar plot under OM. **(E)** Pearson correlations of iSMCs with primary SMCs, without the inclusion of the protein vimentin at days 10 and 17 in NM and OM.

The derived iSMCs were cultured in NM and OM to investigate the potential to calcify. Each of the three differentiations replicates were carried out to days 10 and 17, to ensure calcification under OM. iSMCs treated with OM showed high amounts of calcium deposits after 10 days, and Alizarin Red S staining for calcium deposition was used to measure calcification at day 17. This is represented by bright red color in iSMCs in OM (calcified; c-iSMCs), while iSMCs in NM remain unstained (Figure 7A).

The mean protein expression levels for both endpoints (days 10 and 17) in iSMCs were compared to primary SMCs cultured for 14 days in NM and OM. Representative images of Alizarin Red S staining for calcium deposition of SMCs in both media are shown as well (Figure 7B).

Induced-smooth muscle cells have a relatively higher expression of vimentin under both NM and OM conditions than primary SMCs. Inclusion of the protein vimentin in the correlation analysis had little effect on the Pearson’s correlation of iSMCs and primary SMCs cultured in NM. Pearson’s correlation values in NM were 0.788 and 0.764 with and without the inclusion of vimentin, respectively (Figure 7C). However, inclusion of vimentin had a much greater effect on the Pearson’s correlation values under OM conditions. The Pearson’s correlation between iSMCs and primary SMCs in OM was 0.561 when vimentin was included; however, the exclusion of vimentin caused a Pearson correlation of 0.88 in OM conditions (Figure 7D). When deciding if iSMCs should be cultured in NM or OM for prolonged time periods (17 days vs. 10 days), we show that the Pearson’s correlation at Day 17 is much higher in both NM and OM as compared to Day 10, indicating higher proteomic overlap with primary cells with longer culture duration (Figure 7E). To conclude, iPSC-derived SMCs can be used as a high-fidelity model of primary SMCs.

## Discussion

Intimal calcification is a common outcome of atherosclerosis. It is characterized by the formation of macro- and microcalcifications within the plaque, where microcalcifications contribute to plaque instability. A key cell type in the pathogenesis of atherosclerosis is SMCs, through the secretion of mineralizing EVs that accumulate and form microcalcifications (5, 8, 32–35). Primary SMCs have major limitations that complicate *in vitro* studies, by showing high passage dependency, low proliferation rate and high donor to donor variability (14). To overcome these limitations, iPSC-technology has proven to be a critical alternative, as iPSCs can be grown in large quantities due to their unlimited proliferation potential and ability to differentiate into any desired cell type. This study presented a model for studying SMC contributions in vascular calcification by differentiating iPSCs toward a calcifying SMC phenotype, as seen in vascular calcification.

The protocol for differentiation of iPSC toward iSMC through a lateral plate mesoderm intermediate was based on a previous study from Patsch et al. (16). The protocol was adapted to our cell line; for mesoderm induction BMP4 protein concentration was 10% of their protocol due cell death at higher concentrations. Immunofluorescent staining and flow cytometry analysis of pluripotency markers showed consistency in iPSC pluripotency, with high NANOG and OCT4 expression by immunocytochemistry and positive flow cytometry on markers TRA-1-60 and TRA-1-81.

A limitation of this study is the restriction to one iPS cell line. iPSC lines from different donors, and even clones from the same cell line, have been reported to vary in differentiation potential and efficiency (36, 37). This study utilized iPSC lines derived from foreskin fibroblasts, and thus the iSMCs were male-derived. Female vascular SMCs have been shown to have lower contractility, which could be due to gender-related differences in the expression of estrogen receptors (38). Therefore, the SMCs used for comparison were chosen from male donors to eliminate any confounding differences in the proteome that may be related to sex hormone signaling. For future studies, it is important to test our methods on more iPSC lines to ensure that additional donor cell lines could produce the same results.

The iPSCs were differentiated successfully toward an SMC phenotype within 10 days, showing clear SMC morphology and expression of SMC markers αSMA and MYH11. Flow cytometry analysis showed an efficiency of SMC differentiation of 96% (*n* = 3) CD140b+ cells. The proteome was analyzed throughout the differentiation time course. Cells were collected at days 0 (iPSCs), 3, 5, 7, and 10 (iSMCs). The proteomics data verified a successful iPSC differentiation to iSMCs through a mesodermal intermediate. First, this was shown by an upregulation of mesodermal proteins on day 3 and followed by increasing upregulation of SMC associated proteins at day 5, 7, and 10. Analysis on biological processes showed an initial decrease of pluripotency and increase of mesodermal and other developmental processes. From day 5 to day 10 GO terms associated with muscle development and organizational processes dominated. Finally, Pearson’s correlation coefficient of iSMCs with naïve hCASMCs showed a strong correlation in the expression of SMC marker proteins at days 7 and 10. Together, these results indicate a successful differentiation of iPSCs to iSMCs.

Characterizing SMCs has shown to be challenging and is not universally consistent (10). The most common marker for identification of SMCs is αSMA. However, αSMA is also expressed by other cell types such as myofibroblasts, cardiomyocytes and skeletal muscle cells, and therefore αSMA alone is not enough to define a pure population of SMCs. Other proteins have been described to be elevated in either mature SMCs or during the differentiation of SMCs, including CNN1, smooth muscle protein 22 alpha, myosin light chains, transgelin, vinculin, vimentin, desmin, tropomyosin, and CD140b. However, these markers are not specific for SMCs either. The most specific markers for mature SMCs currently known are smoothelin and MYH11, but these proteins are also dependent on SMC maturity. Due to this, characterization of SMCs is limited and fails to exclude the outcome of having cell types that show high resemblance with SMCs such as myofibroblasts (39). Because of possible mischaracterization, it is unclear whether previous studies show a pure SMC population. The extensive amount of data provided by proteomics analysis in our study enables higher certainty of proving purity of the iSMC population. This introduces a method to research the course of SMC differentiation and possibly identify novel factors required for SMC transition.

After iSMC differentiation, we initiated calcification in the iSMCs by culturing the cells in osteogenic media for up to 17 days. Alizarin Red S staining verified successful transformation of iSMCs into c-iSMCs. Proteomic analysis showed increase of Annexin 5 in c-iSMCs (data not shown). Annexin 5 has been demonstrated to play an important role in the generation of microcalcifications (40). It contributes in pathological extracellular vesicle mineralization by facilitating hydroxyapatite nucleation (41). Kegg pathway carbon metabolism, and GO terms for various metabolic processes, vesicle mediated transport and phosphate regulatory processes show up as functional enrichments in the network of proteins. These terms and pathways have previously been associated with calcification (42–44). Biological processes, which appear low in c-iSMCs included developmental processes and intracellular organizational processes such as adherens junctions, supramolecular fiber, collagen fiber and cytoskeleton organization. This indicates a less organized, less mature, more plastic and less contractile phenotype, as seen in a more synthetic (calcifying) SMC phenotype (10). More functional assays assessing contractility and proliferative capacity would help to further evaluate iSMC functional plasticity, which was not addressed herein.

Correlation analysis between the generated iSMCs cultured under NM or OM conditions showed high proteomic overlap with primary SMCs cultured under the same conditions. However, regardless of media conditioning, iSMCs showed a persistently high vimentin protein expression. When cultured in NM, the inclusion of vimentin in the correlation analysis had little effect and actually lowered the correlation coefficient when removed. In contrast, inclusion of vimentin in the correlation analysis under osteogenic media conditions had a much larger effect on the correlation coefficient, raising it from 0.561 to 0.88 upon removal. The persistence of the high vimentin protein expression may be a hallmark of iPSC-derived cell lines and should be considered in future studies. Previous differentiation experiments of iPSC-derived erythroid cells also resulted in a persistent vimentin expression that impeded enucleation (45). Other studies have shown that dedifferentiation during reprogramming may be associated with cytoskeleton remodeling to a more rudimentary state that persists even after iPSC differentiation (46). Future studies should take into consideration the possibility of 3-dimensional cell culture using hydrogels. A more physiologically relevant culture condition that recreates an *in vivo*-like microenvironment may lead to even higher overlap between iSMC ECM protein expression levels and provide a higher fidelity model.

Studying the role of SMCs during calcification using “omics” approaches is becoming more common; however, these studies are usually limited to diseased tissue and appropriate, non-diseased controls are typically lacking. Oftentimes, the initiating events of calcification are missed in tissue that has already reached an advanced disease stage by the time of diagnosis. While it is possible to identify disease-drivers at this stage, it is most likely an accumulation of several confounding metabolic events instead of initiating factors. Therefore, the premise of pharmacological intervention requires bridging the gap between disease initiation and clinical presentation. The combination of an iPSC-derived model of SMC calcification and “omics” analysis may help to identify the starting factors involved in calcification while deconvoluting the pathways involved in the end-stage disease (47). This study presents an inexhaustible source of functional iSMCs and calcifying iSMCs to create vascular calcification tissue model systems, which holds a high potential for future applications in cardiovascular calcification research and drug discovery.

## Data availability statement

The mass spectrometry proteomics data have been deposited to the ProteomeXchange Consortium via the PRIDE partner repository with the dataset identifier PXD032353.

## Author contributions

SA and AS contributed equally to the manuscript. All authors listed have made a substantial, direct, and intellectual contribution to the work, and approved it for publication.

## Conflict of interest

The authors declare that the research was conducted in the absence of any commercial or financial relationships that could be construed as a potential conflict of interest.

## Publisher’s note

All claims expressed in this article are solely those of the authors and do not necessarily represent those of their affiliated organizations, or those of the publisher, the editors and the reviewers. Any product that may be evaluated in this article, or claim that may be made by its manufacturer, is not guaranteed or endorsed by the publisher.

## Funding

MA and EA were supported by research grants from the National Institutes of Health (R01HL126901 and R01HL149302 to MA; R01HL136431, R01HL141917, and R01HL147095 to EA) and Kowa Company, Ltd, Nagoya, Japan (A11014 to MA). The funders played no role in the design, data collection, and analysis of the studies described here, or in preparation of the present manuscript.

## Supplementary material

The Supplementary Material for this article can be found online at: https://www.frontiersin.org/articles/10.3389/fcvm.2022.925777/full#supplementary-material

Supplementary Figure 1 Proteomics analysis. **(A)** Snapshot of overall proteomics data divided based on highest protein abundance on each day. **(B)** Z-score Abundance (eq. 1) vs. various threshold to obtain the threshold = 1.5 showing multiplicity of 1. **(C)** Number of Day-specific proteins on each day.

## Footnotes

1
https://bioconductor.org/packages/release/bioc/html/XINA.html

2
https://www.uniprot.org/uniprot/Q53F18
