# Supplementary material for: Induced pluripotent stem cell-derived smooth muscle cells to study cardiovascular calcification
Source: Front Cardiovasc Med. 2022 Jul 22;9:925777. doi: 10.3389/fcvm.2022.925777 (PMC9357895; doi:10.3389/fcvm.2022.925777)
Supplement: Supplementary file 1 [file Data_Sheet_1.DOCX]

## hiPSC - iVSMC differentiation protocol:

Based on Patsch, et al., 2015

**Timeline**

*Synthetic SMCs differentiation*

**Day 8 – 10**Purification

**Day 5**N2B27
PDGF-BB

**Day 3**
N2B27
PDGF-BB
Activin A

**Day 0**
N2B27
CHIR99021
BMP4

**Day -1**
iPSC passage

*Lateral mesoderm induction*

**Plates set up example:**24 well plates: 14 wells
12 wells plates: 6 wells
6 well plates: 12 wells
Seeding density: 37.000 – 47.000 cells/cm^2^

**Approximately 7x 10^6^ iPSCs needed per protocol**

**Day -1:**

1. Prepare N2B27 media:

250mL DMEM/F12 medium

250mL Neurobasal medium

10mL B27 (1.94%)

5ml N2 (0.97%)

0.5ml β-Mercaptoethanol (0.097%)

5mL P/S (1%)

- store at 4°C up to 1 month

1. Matrigel coat plates
   (in dept protocol coating can be found in iPSC protocol)

2x 7 wells of 24 wells plate: 0.25 mL per well

2x 3 wells of 12 wells plate: 0.5 mL per well
2 6 wells plates = 12 mL

Dissolve 120uL aliquot in 19mL ice cold DMEM/F12🡪 concentration 68.21 ug/mL 🡪 use this
Incubate for at least 1 hour
*Note: Make sure that the full surface of the well is covered!*

1. Prepare mTeSR + 0.1% ROCK inhibitor media
2. Passage iPS cells (see iPSC protocol):
   Use media prepared in previous step
   Make single cell suspension by resuspending thoroughly with p1000 or even with p200.
   Count cells (when adding 10uL to the small tube, resuspend this 10uL thoroughly to make sure its single cell, since the cell counter is affected by aggregates)
   Seed at density stated above:
   1. 24 wells plates:
      80,000 cells per well
      make concentration of 160,000 cells/mL 🡪 feed 0.5mL per well
   2. 12 wells plate:

140,000 cells per well
make concentration of 140.000 cells/mL 🡪 feed 1 mL per well

- 1. 6 wells plates:
     400.000 cells per well

Make concentration of 200,000 cells/mL 🡪 feed 2 mL per well

**Day 0:**

1. prepare media N2B27 + 8um CHIR-99021 + 2.5ng/mL BMP4
   1. CHIR-99021: stock 10mM 🡪 0.8uL/mL media. Explained: 80% of 1:1000 of end volume (so if you make 100mL, 80uL of CHIR)
   2. BMP4: stock 2.5ug/mL 🡪 1uL/mL media (1:1000)
      (note: Patsch protocol does 25ng/mL, we do 2.5ng/mL)
2. Double feed cells

*Example math media:
2 6 wells plates: 4 mL per well 🡪 2 x 6 x 4 = 48
2 24 wells plates of 7 wells: 1 mL per well 🡪 2 x 7 x 1 = 14
2 12 wells plates of 3 wells: 2 mL per well 🡪 2 x 3 x 2 = 12
Total mL needed: 48+14+12 = 72.
🡪 make 80 mL
= 80mL N2B27 + 64uL CHIR + 80uL BMP4*

**Leave for three days without media change!**

**Day 3:**

1. Prepare media: 1.5 feed
   N2B27 + 10 ng/mL PDGF + 2ng/mL ActivinA
   1. PDGF-BB: stock 10ug/mL 🡪 1 uL/mL media 🡪 1:1000
   2. ActivinA: stock 2ug/mL 🡪 1uL/mL media 🡪 1:1000

*Example math media:
2 6 wells plates: 3 mL per well 🡪 2 x 6 x 3
2 24 wells plates of 7 wells: 0.75 mL per well 🡪 2 x 7 x 0.75
2 12 wells plates of 3 wells:1.5 mL per well 🡪 2 x 3 x 1.5
Total mL needed: 55.5 mL
🡪 double amount because media change also done on day 4.
🡪 make 120 mL + 120uL PDGF-BB + 120uL ActivinA*

1. Timepoint D3
   Wash wells with PBS without calcium and magnesium
   24 wells plate
   1. 3 wells 🡪 collect in 100uL RIPA 🡪 store at -80
   2. 4 wells 🡪 fix in PFA 🡪 stain for pluripotency and SMC markers

12 wells plate

- 1. 3 wells 🡪 collect in 1mL trizol 🡪 store at -80

**Day 4**: media change – media prepared on day 3

**Day 5**: Replate

1. Timepoint

Wash wells with PBS without calcium and magnesium
24 wells plate

- 1. 3 wells 🡪 collect in 100uL RIPA 🡪 store at -80
  2. 4 wells 🡪 fix in PFA 🡪 stain for pluripotency and SMC markers

12 wells plate

- 1. 3 wells 🡪 collect in 1mL Trizol 🡪 store at -80

1. Passage cells with trypsin
2. Replate at 30,000 cells/cm^2^24 wells plate: 57,000 cells/well
   12 wells plate: 105,000 cells/well
   6 wells plate: 288,000 cells/well

| Synthetic protocol  Media: N2B27 + 10ng/mL PDGF-BB Coating: gelatin  *Example media:*  *Make media for media change d5 and d7 example 120mL + 120uL PDGF-BB* | Contractile protocol  Media: N2B27 + 2ng/mL ActivinA + heparin (2ug/mL) Coating = collagen  *Example media:*  *Make media for media change d5 and d7*  *Example 120mL + 120uL ActivinA + 120uL Heparin* |
| --- | --- |

Media preparation: media change on day 5 and day 7 so make for 2 media changes. Amount of plates is dependent on outcome of experiment.

**Day 7**: timepoint + media change

**Day 10:** timepoint + end point
