# Supplementary figures and images for: Induced pluripotent stem cell-derived smooth muscle cells to study cardiovascular calcification
Source: Front Cardiovasc Med. 2022 Jul 22;9:925777. doi: 10.3389/fcvm.2022.925777 (PMC9357895; doi:10.3389/fcvm.2022.925777)

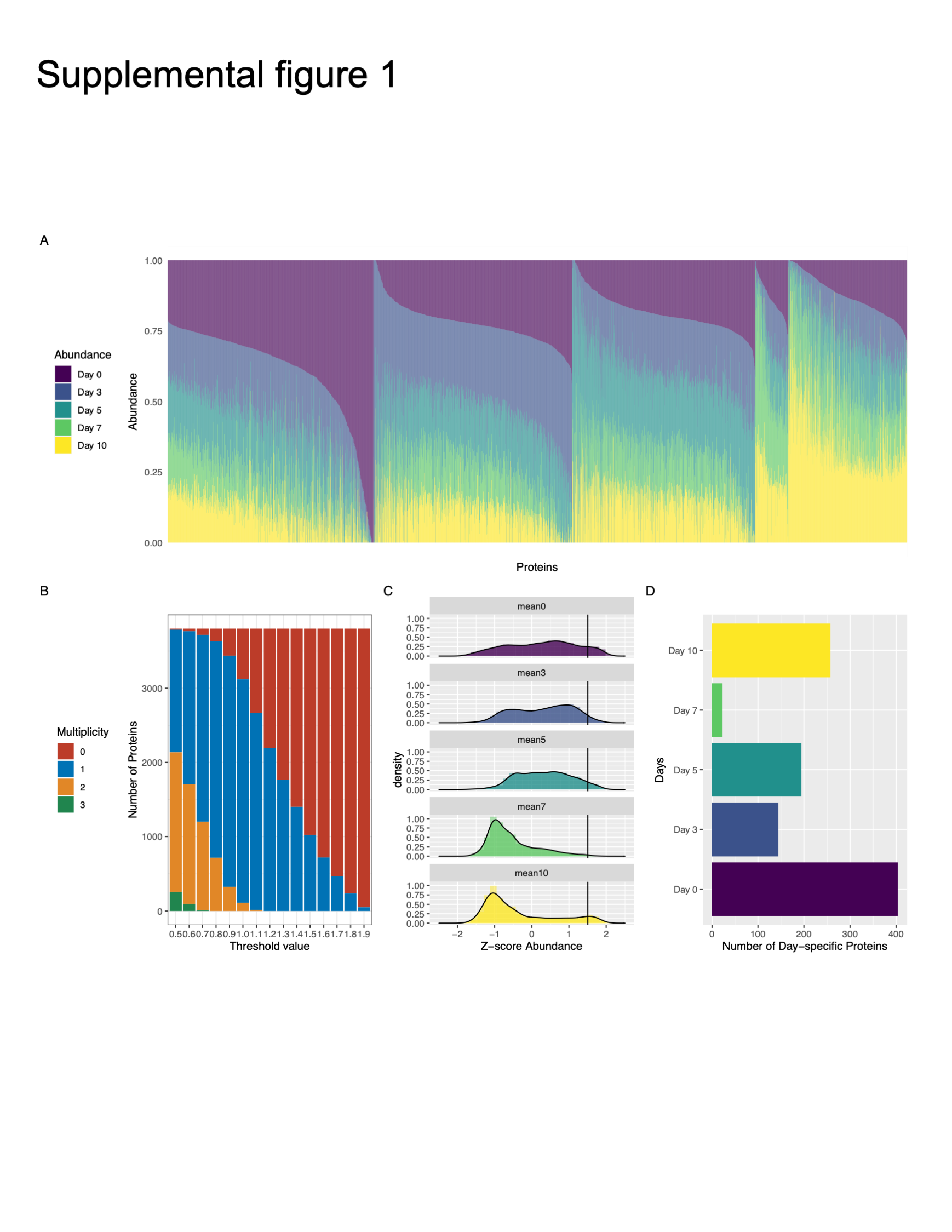

Supplement: Supplementary Figure 1 — Proteomics analysis. (A) Snapshot of overall proteomics data divided based on highest protein abundance on each day. (B) Z-score Abundance (eq. 1) vs. various threshold to obtain the threshold = 1.5 showing multiplicity of 1. (C) Number of Day-specific proteins on each day. [file Image_1.TIFF]
